# Frederick W. Alt received the 2015 Szent-Györgi Prize for Progress in Cancer Research

**DOI:** 10.1186/s40880-015-0075-x

**Published:** 2016-02-03

**Authors:** Peter Scully, Jie Zhao, Sujuan Ba

**Affiliations:** National Foundation for Cancer Research, Bethesda, MD USA

**Keywords:** The National Foundation for Cancer Research, The Szent-Györgyi Prize, Frederick Alt, Gene amplification, Non-homologous end joining

## Abstract

The Szent-Györgyi Prize for Progress in Cancer Research is a prestigious scientific award established by the National Foundation for Cancer Research (NFCR)—a leading cancer research charitable organization in the United States that is committed to supporting scientific research and public education relating to the prevention, early diagnosis, better treatments, and ultimately, a cure for cancer. Each year, the Szent-Györgyi Prize honors an outstanding researcher, nominated by colleagues or peers, who has contributed outstanding, significant research to the fight against cancer, and whose accomplishments have helped improve treatment options for cancer patients. The Prize also promotes public awareness of the importance of basic cancer research and encourages the sustained investment needed to accelerate the translation of these research discoveries into new cancer treatments. This report highlights the pioneering work led by the 2015 Prize winner, Dr. Frederick Alt. Dr. Alt’s work in the area of cancer genetics over four decades has helped to shape the very roots of modern cancer research. His work continues to profoundly impact the approaches that doctors around the globe use to diagnose and treat cancer. In particular, his seminal discoveries of gene amplification and his pioneering work on molecular mechanisms of DNA damage repair have helped to usher in the era of genetically targeted therapy and personalized medicine.

In recognition for his pioneering work in the area of cancer genetics, Dr. Frederick Alt was awarded the 2015 Szent-Györgyi Prize for Progress in Cancer Research. Dr. Alt’s research is foundational to the modern understanding of cancer as a genetic disease, and has led directly to the era of precision medicine. The Prize was presented at a ceremony in Washington, D.C., on April 29, 2015, at The National Press Club.

The Szent-Györgyi Prize for Progress in Cancer Research is a prestigious scientific award established by the National Foundation for Cancer Research (NFCR)—a leading cancer research charitable organization in the United States that has gained international recognition for its vision and unique approaches to accelerating cancer research toward a cure. The Prize is awarded annually to a scientist, nominated by colleagues or peers, who has contributed outstanding, significant research to the fight against cancer, and whose accomplishments have helped improve treatment options for cancer patients.

The Prize is named in honor of NFCR co-founder Albert Szent-Györgyi, M.D., Ph.D., who won the Nobel Prize for Science and Medicine in 1937 for his discovery of vitamin C. The Prize and the surrounding ceremony also serve to promote public awareness of the importance of basic cancer research and to encourage the sustained investment needed to accelerate the translation of these research discoveries into new cancer treatments.

Since its establishment in 2006, the Szent-Györgyi Prize has been awarded to 11 outstanding cancer researchers from around the world. Among the past awardees is Dr. Zhu Chen, chairman of the Chinese Medical Association and former Minister of Health of China, who won the 2012 Prize together with his mentor, Dr. Zhen-Yi Wang.

## The 2015 Szent-Györgyi Prize

**Fig. 1:**
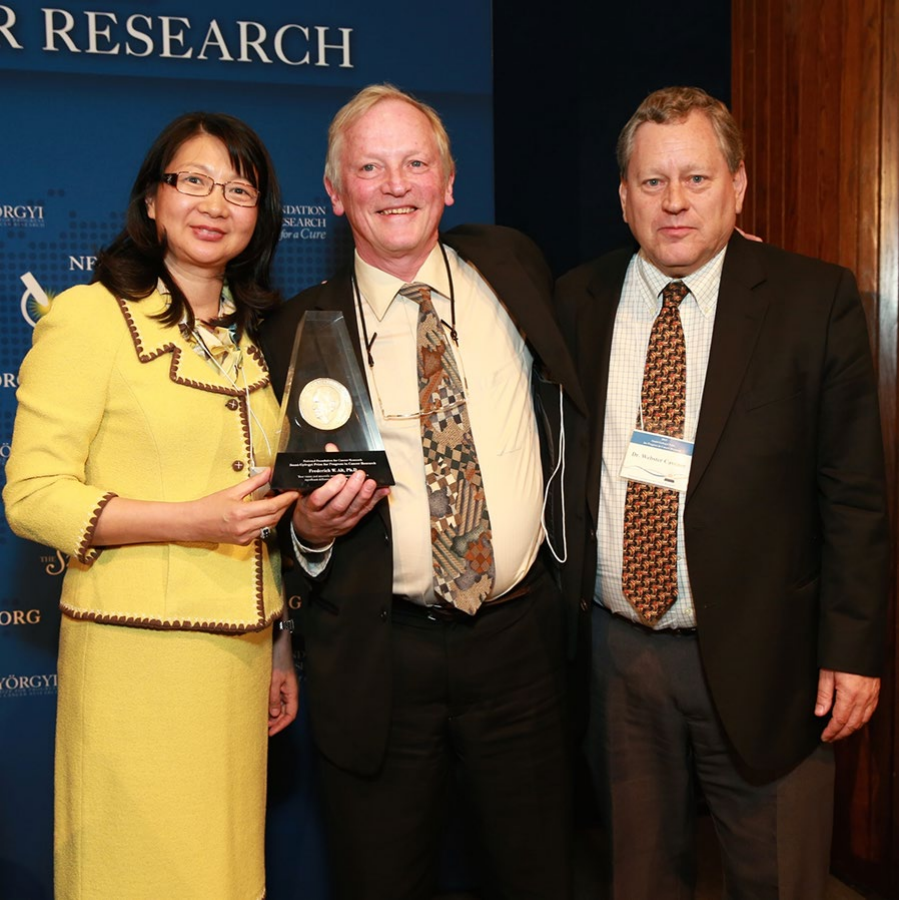
*From left to right* Dr. Sujuan Ba, Co-chair of the 2015 Szent-Györgyi Prize Selection Committee and President of the National Foundation for Cancer Research (NFCR); Dr. Frederick Alt, winner of the 2015 Szent-Györgyi Prize; and Dr. Webster Cavenee, winner of the 2007 Szent-Györgyi Prize

In 2015, the Szent-Györgyi Prize for Progress in Cancer Research was awarded to Frederick Alt, Ph.D., Professor of Genetics at Harvard Medical School, Director of the Program in Cellular and Molecular Medicine at Boston Children’s Hospital, and Howard Hughes Medical Institute Investigator (Fig. 1). Dr. Alt’s groundbreaking work in cancer genetics over four decades has helped to shape the very roots of modern cancer research. Today, that work continues to bear fruit, profoundly impacting the approaches that doctors use to diagnose and treat cancer.

NFCR’s selection committee was unanimous in its decision to recognize Dr. Alt, whose work has proven foundational to the modern understanding of cancer—not only how the lethal disease forms but also how it can become resistant to treatment. In particular, his seminal discoveries of gene amplification and his pioneering work on molecular mechanisms of DNA damage repair have helped to usher in the era of genetically targeted therapy and personalized medicine.

“Dr. Alt’s work has uncovered and explained some of the most foundational chemistry of life, and throughout his career he has always been focused on the implications for cancer,” said Sujuan Ba, Ph.D., Co-chair of the 2015 Szent-Györgyi Prize Selection Committee and President of NFCR. “His vision and talent were instrumental in bringing cancer research into the modern era, and we are proud to present him with this award.”

In bestowing this award, the Prize Selection Committee recognizes the entire body of work that Dr. Alt has amassed. “Dr. Alt is a consistently outstanding scientist throughout his career,” said Dr. James Allison, Executive Director of the Immunotherapy Platform at MD Anderson Cancer Center, winner of the 2014 Szent-Györgyi Prize, and Chair of the 2015 Prize Selection Committee. “The genetic processes Dr. Alt described are central to understanding the mechanisms that cause cancer, and have ultimately led to an entire class of targeted therapy and associated diagnostics that are providing benefit to countless cancer patients.”

## Frederick W. Alt: cancer genetics pioneer

Dr. Frederick Alt is currently the Director of the Program in Cellular and Molecular Medicine (formerly the Immune Disease Institute) at Boston Children’s Hospital. Dr. Alt has been Professor of Genetics at Harvard Medical School since 1991 and Charles A. Janeway Professor of Pediatrics at Boston Children’s Hospital since 1993. Prior to 1991, Dr. Alt was on the Faculty at Columbia University. He earned his Ph.D. with distinction from the Department of Biological Sciences at Stanford University in 1977. Dr. Alt is a member of the National Academy of Sciences, the Institute of Medicine of the National Academies, and a fellow of the American Academy of Arts and Sciences, the American Academy of Microbiology, and the American Association for the Advancement of Science. He is also an investigator at the Howard Hughes Medical Institute.

Dr. Alt has won numerous honors for biomedical research, including the Stanford University Medical Center Arthur Kornberg and Paul Berg Lifetime Achievement Award in Biomedical Science and the National Cancer Institute’s Alfred Knudson Award for “pioneering contributions that have revolutionized the field of cancer genetics.” Each year, the Cancer Research Institute of New York presents the Frederick W. Alt Award for New Discoveries in Cancer Immunology in his honor. Dr. Alt’s impact extends far beyond his own laboratory, for not only is he an outstanding researcher; he is also an exemplary teacher. In 2003, he received the American Association of Immunologists Excellence in Mentoring Award. Dr. Alt has mentored over 100 students and research fellows, many of whom have become leaders in immunology, genetics, or cancer biology.

**Fig. 2:**
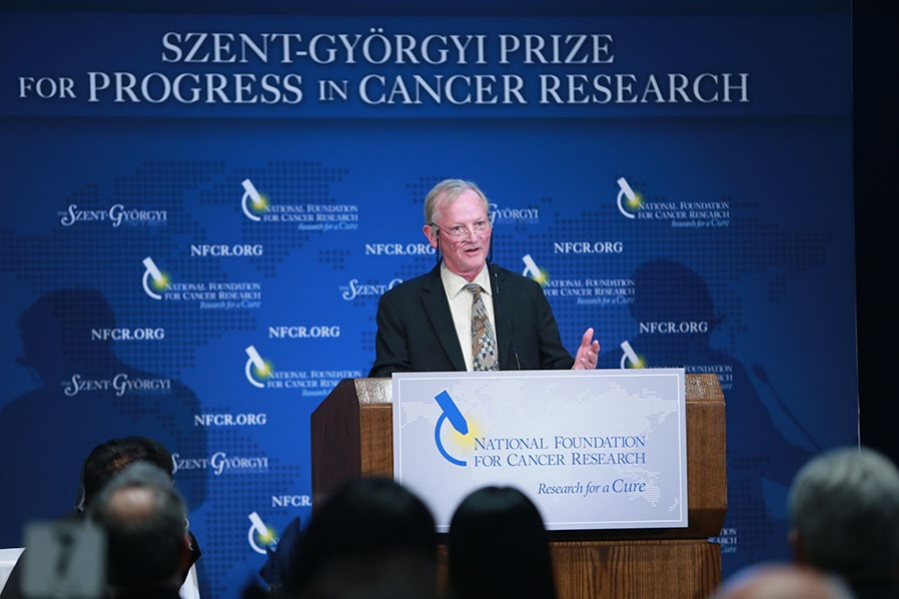
Dr. Frederick Alt, winner of the 2015 Szent-Györgyi Prize, gave an acceptance speech at the Award Ceremony held on April 29, 2015 in Washington D.C.

For Dr. Alt, cancer research has always been a personal endeavor. During his remarks, Dr. Alt spoke of how cancer claimed the lives of both his parents when he was a young child (Fig. 2). “It did make a big impact,” said Dr. Alt, “and I wanted to become a cancer researcher.”

Drug resistance was a well-known problem in cancer treatment when Dr. Alt’s career began. His early research sought to understand how cancer cells become resistant to the drug methotrexate, and it was animated by the “somewhat heretical notion that cancer cells might be able to increase their numbers of gene copies.” At that time, in the early 1970s, conventional wisdom held that each person had exactly two copies of each gene—one copy from their mother and the other from their father—and that their genome was otherwise fixed and unchangeable.

After building the technology to probe cells for individual genes, Dr. Alt applied this technology to cancer cells that had been treated with methotrexate. “We found that cells that are very resistant have hundreds or thousands of copies of the dihydrofolate reductase gene which confers resistance,” said Dr. Alt. “When we took the cells out of the drug, they actually lost the gene copies, really showing that mammalian cancer cells could have unstable genomes. They could gain genes when they were put under selection for drug resistance, and they could lose them when it was taken away. That was really a shocking result.”

Dr. Alt’s discovery of gene amplification in chemotherapy-resistant cancer cells served to overthrow the old concept of a fixed genome, suggesting instead that cancer cells could change their genes to evolve resistance to treatments like methotrexate.

The story became even more interesting when the same pattern of amplified genes was discovered in the childhood cancer neuroblastoma. “We knew from our methotrexate resistance research what amplified genes looked like under a microscope,” said Dr. Alt, but it was not clear which gene had been amplified in these tumors. It was at this time that Dr. Alt made the connection between gene amplification and carcinogenesis. “The idea we had was that there would be a gene that actually caused the cancer—not one that would cause drug resistance, but one that made the cancer grow better,” he said. This idea built directly off the recent discovery of viral oncogenes by previous Szent-Györgyi Prize winner Dr. Peter Vogt.

Dr. Alt and his colleagues identified that the amplified gene in neuroblastoma was related to the viral oncogene *myc*, so they named it neuroblastoma-myc or *n*-*myc*. “That was extremely satisfying for us,” said Dr. Alt, “because it showed that for gene amplification it was not only a mechanism by which cancer cells become drug-resistant but also a mechanism by which they get generated.” Today, genomic instability is recognized as one of the hallmarks of cancer.

Equally important to cancer research has been Dr. Alt’s work on the critical DNA repair mechanism called “non-homologous end joining” (NHEJ). Dr. Alt not only made the initial experimental findings that led to the discovery of this pathway but also carried out an ingenious series of experiments over many years in his lab in Boston, taking it apart piece by piece to understand how it works. This work linked NHEJ to protecting against a specific type of DNA damage called translocation, which is a major component of many cancers, especially leukemia and lymphoma.

Both amplified genes and translocated genes are key components of the Precision Medicine paradigm, which is at the heart of 21st century medicine. By identifying the source of genetic abnormalities that drive both cancer development and drug resistance, Dr. Alt’s insights helped to revolutionize cancer diagnostics and treatment. His discoveries led to a wholly new approach to treating cancer—identifying these genetic abnormalities, and then selecting new drugs that target each specifically.

In his closing remarks at the award ceremony, Dr. Alt said “Now is the time. We have the opportunity, and if we get the support I’m confident that basic cancer-driven research will help exploit this current technological explosion, [producing] more and more breakthroughs at an ever-increasing pace.” Through events like the Szent-Györgyi Prize, NFCR is working towards this goal, by raising public awareness of research and encouraging sustained investment in the future of basic science.

*The 2015 Szent*-*Györgyi Prize Selection Committee was chaired by James Allison, Ph.D., and co*-*chaired by Sujuan Ba, Ph.D. Other selection committee members included leaders in cancer research and drug development from academic institutes and biotech and pharmaceutical industries: Webster K. Cavenee, Ph.D., Ludwig Institute for Cancer Research; Carlo M. Croce, M.D., The Ohio State University; George D. Demetri, M.D., Dana*-*Farber Cancer Institute; Richard Gaynor, M.D., Eli Lilly and Company; Thomas J. Kelly, M.D., Ph.D., Memorial Sloan*-*Kettering Cancer Center; Mary*-Claire King, Ph.D., University of Washington School of Medicine; Scott M. Lippman, M.D., Moores Cancer Center; Tak W. Mak, Ph.D., Ontario Cancer Institute; Alex Matter, M.D., Experimental Therapeutics Center & D3, A*STAR Singapore; Scott D. Patterson, Ph.D., Amgen, Inc.; Philip Tsichlis, M.D., Tufts University School of Medicine; Peter K. Vogt, Ph.D., The Scripps Research Institute; Owen N. Witte, M.D., David Geffen School of Medicine, University of California, Los Angeles; Qimin Zhan, M.D., State Key Laboratory of Molecular Oncology, China; and General Secretary Yi Michael Want, M.D., Ph.D., NFCR.

## Previous recipients of the Szent-Györgyi Prize for Progress in Cancer Research

2014—James Allison, Ph.D., Chairman of the Immunology Department and Executive Director of the Immunotherapy Platform for the “Moon Shots” Program, University of Texas MD Anderson Cancer Center, Houston, TX, US.

2013—Alex Matter, M.D., Chief Executive Officer, Experimental Therapeutics Centre, Agency for Science, Technology and Research (A*STAR), Singapore.

2012—Zhu Chen, M.D., Ph.D., Professor, School of Medicine of Shanghai Jiao Tong University; Chairman, Chinese Medical Association; former Minister of Health of China, Shanghai, P.R. China.

2012—Zhen-Yi Wang, M.D., Professor, School of Medicine of Shanghai Jiao Tong University; Honorary Director, Shanghai Institute of Hematology, Shanghai, P.R. China.

2011—Beatrice Mintz, Ph.D., Professor and Jack Schultz Chair in Basic Science, Fox Chase Cancer Center, Philadelphia, PA, US.

2010—Peter K. Vogt, Ph.D., Professor, Department of Molecular and Experimental Medicine, The Scripps Research Institute, La Jolla, CA, US.

2009—Ronald A. DePinho, M.D., President, University of Texas MD Anderson Cancer Center, Houston, TX, US.

2008—Carlo M. Croce, M.D., Director, Human Cancer Genetics Program; Director, Institute of Genetics, The Ohio State University, Columbus, OH, US.

2007—Webster K. Cavenee, Ph.D., Director, Ludwig Institute for Cancer Research, San Diego Branch; Distinguished Professor, University of California, San Diego, CA, US.

2006—Harold F. Dvorak, M.D., Mallinckrodt Professor Emeritus of Pathology, Harvard Medical School; Chief, Department of Pathology, Beth Israel Deaconess Medical Center, Boston, MA, US.

